# CCBE1 Is Essential for Epicardial Function during Myocardium Development

**DOI:** 10.3390/ijms232012642

**Published:** 2022-10-20

**Authors:** Fernando Bonet, Sabrina Brito Añez, José Manuel Inácio, Matthias E. Futschik, José Antonio Belo

**Affiliations:** 1 Stem Cells and Development Laboratory, iNOVA4Health, NOVA Medical School/Faculdade de Ciências Médicas, Universidade NOVA de Lisboa, 1169-056 Lisbon, Portugal; 2 Medicine Department, School of Medicine, University of Cádiz (UCA), 11003 Cádiz, Spain; 3 MRC London Institute of Medical Sciences (LMS), Faculty of Medicine, Hammersmith Hospital Campus, Imperial College London, Du Cane Road, London W12 0NN, UK

**Keywords:** CCBE1, heart development, epicardium, epicardial derived cells, EMT, myocardial growth

## Abstract

The epicardium is a single cell layer of mesothelial cells that plays a critical role during heart development contributing to different cardiac cell types of the developing heart through epithelial-to-mesenchymal transition (EMT). Moreover, the epicardium is a source of secreted growth factors that promote myocardial growth. CCBE1 is a secreted extracellular matrix protein expressed by epicardial cells that is required for the formation of the primitive coronary plexus. However, the role of CCBE1 during epicardial development was still unknown. Here, using a *Ccbe1* knockout (KO) mouse model, we observed that loss of CCBE1 leads to congenital heart defects including thinner and hyper-trabeculated ventricular myocardium. In addition, *Ccbe1* mutant hearts displayed reduced proliferation of cardiomyocyte and epicardial cells. Epicardial outgrowth culture assay to assess epicardial-derived cells (EPDC) migration showed reduced invasion of the collagen gel by EPDCs in *Ccbe1* KO epicardial explants. *Ccbe1* KO hearts also displayed fewer nonmyocyte/nonendothelial cells intramyocardially with a reduced proliferation rate. Additionally, RNA-seq data and experimental validation by qRT-PCR showed a marked deregulation of EMT-related genes in developing *Ccbe1* mutant hearts. Together, these findings indicate that the myocardium defects in *Ccbe1* KO mice arise from disruption of epicardial development and function.

## 1. Introduction

Heart development is a finely orchestrated process that gives rise to the morphologically mature heart, with four cardiac chambers, thickened ventricular walls, fully formed atrioventricular valves, septated outflow tract, and a functional coronary vasculature system. One critical morphogenetic process during heart development is myocardial compaction, which is essential for generating a functionally competent ventricular wall [1]. Defects of ventricular wall compaction (noncompaction) are closely related to left ventricular noncompaction (LVNC), a type of inherited cardiomyopathy [2]. At early stages, the embryonic heart is formed by two concentric layers, a thin outer myocardium, and an inner endocardium [1]. At embryonic day, (E)9.0, trabecular ridges emerge, whereby myocardial projections extend initiating trabeculation [1]. Further expansion of the primitive trabecular myocardium (between E9.5 and E13.5) generates the mature trabeculae, which facilitates oxygen and nutrient exchange in the heart muscle [1]. Later, from E14.5, the trabecular myocardium starts to collapse at its base gradually integrating into the ventricular wall thickness forming the compact myocardium, which is destined to form the ventricular wall that provides the bulk of the force of ventricular contraction [3].

The epicardium is a single cell layer of mesothelial cells that covers the outer surface of the heart (E9.5–E10.5) [4,5]. Decades of research have revealed that the epicardium plays a crucial role during heart development, contributing to distinct cardiac cell linages. These include pericytes, coronary endothelial, and smooth muscle cells, which are critical for cardiac coronary vessel formation, as well as cardiac fibroblasts [6,7,8]. Approximately at E12.5 in mice, the epicardium initiates an epithelial-to-mesenchymal transition (EMT) detaching from the epicardial layer and migrating first into the subepicardial space giving rise to the epicardial-derived cells (EPDC). Then, these EPDCs migrate intramyocardially, invading the myocardial wall, and differentiate into distinct cardiac cell lineages [8]. In addition, numerous studies have shown that the epicardium is required for the ventricular wall through EPDC-cardiomyocyte crosstalk, as its genetic or surgical ablation resulted in an underdeveloped compact zone and myocardial growth disruption [9]. Despite its role in heart development, the epicardium is also involved in cardiac regeneration under the stress of injury by secreting growth factors and differentiating to cardiac lineage cells [10,11,12,13,14]. Identification of signaling pathways that regulate epicardial cell behavior will provide insights into the complex regulation of heart development.

The collagen- and calcium-binding EGF-like domains 1 (CCBE1) is an extracellular matrix (ECM) protein described to be essential for lymphatic vascular development via promoting vascular endothelial growth factor C (VEGFC) proteolysis [15,16,17,18]. In our previous work, we unveiled a new role of CCBE1 in coronary vasculature development [18]. We identified *Ccbe1* expression in the epicardium and sinus venosus myocardium at the stage in which coronary vessels start to form (E11.5–E13.5) [18]. Accordingly, *Ccbe1* mutant mice (*Ccbe1^tm1Lex^*) displayed defective coronary vascularization suggesting a key role of CCBE1 for proper coronary vasculature formation [18].

Here, we discovered that, besides coronary defects, CCBE1 loss-of-function also leads to disruption in ventricular growth, and disturbance of epicardial development. *Ccbe1^tm1Lex^* hearts displayed thinner compact myocardium and decreased cardiomyocyte and epicardial cell proliferation. Moreover, in vitro assays showed reduced epicardial migration in *Ccbe1^tm1Lex^* epicardial explants. Transcriptome analysis of mRNA in ventricles from *Ccbe1^tm1Lex^* hearts showed deregulation of EMT- and coronary endothelial-related genes, ultimately supporting that CCBE1 also plays an important role in the epicardial contribution to the developing heart.

## 2. Results

### 2.1. CCBE1 Is Required for Growth and Development of Ventricular Wall

**Figure 1:**
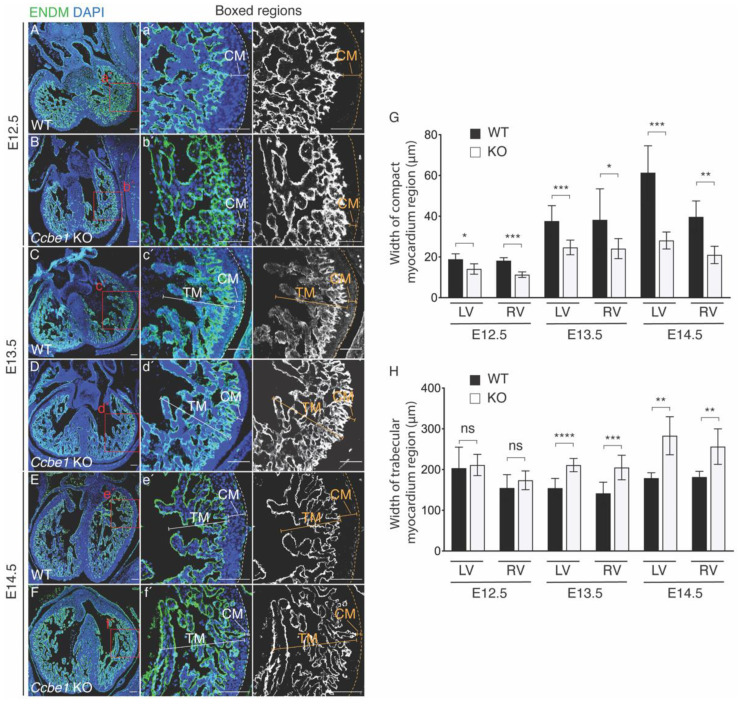
*Ccbe1^tm1Lex^* mice display thinner right and left ventricle wall. (**A**–**F**) ENDM immunofluorescence in transverse sections of E12.5 (**A**,**B**), E13.5 (**C**,**D**), and E14.5 (**E**,**F**) hearts. Images are representative of the following number of replicates: wild-type, *n* = 4; mutant, *n* = 5 at E12.5; wild-type, *n* = 7; mutant, *n* = 8 at E13.5; wild-type, *n* = 5; mutant, *n* = 5 at E14.5. (**a′**–**f′**) are magnification of the LV region indicated by the boxed areas in panels (**A**–**F**), respectively. (**G**) Compact myocardium is thinner in right and left ventricle of *Ccbe1* mutant hearts at E12.5, E13.5 and E14.5 stages. (**H**) Trabecular myocardium thickness is increased in the left and right ventricles in *Ccbe1* mutant hearts at E13.5 and E14.5 stages. ns, nonsignificant; * *p* < 0.05; ** *p* < 0.01; *** *p* < 0.001; **** *p* < 0.0001. Differences were considered statistically significant when *p* < 0.05. Error bars represent SDs. Scale bars = 100 μm. RV, right ventricle; LV, left ventricle.

Previously, we reported that *Ccbe1* mutant hearts exhibit defective coronary vasculature development [18]. Here, to further investigate the impact of CCBE1 ablation in the developing heart, we conducted a detailed study of *Ccbe1^tm1Lex^* cardiac phenotype. Histological analysis using *endomucin* (ENDM) immunofluorescence to highlight the endocardium, shows that the compact myocardium is markedly thinner in both the left and right ventricle of *Ccbe1^tm1Lex^* hearts from E12.5 to E14.5 compared to that in wild-type hearts (Figure 1A–G). In addition, *Ccbe1^tm1Lex^* hearts displayed abnormally large trabecular myocardium in both ventricles at E13.5 and E14.5 as compared to wild-type hearts (Figure 1C–F,H). No other obvious defects were detected in the heart at these stages, which is consistent with previous reports [19,20].

**Figure 2:**
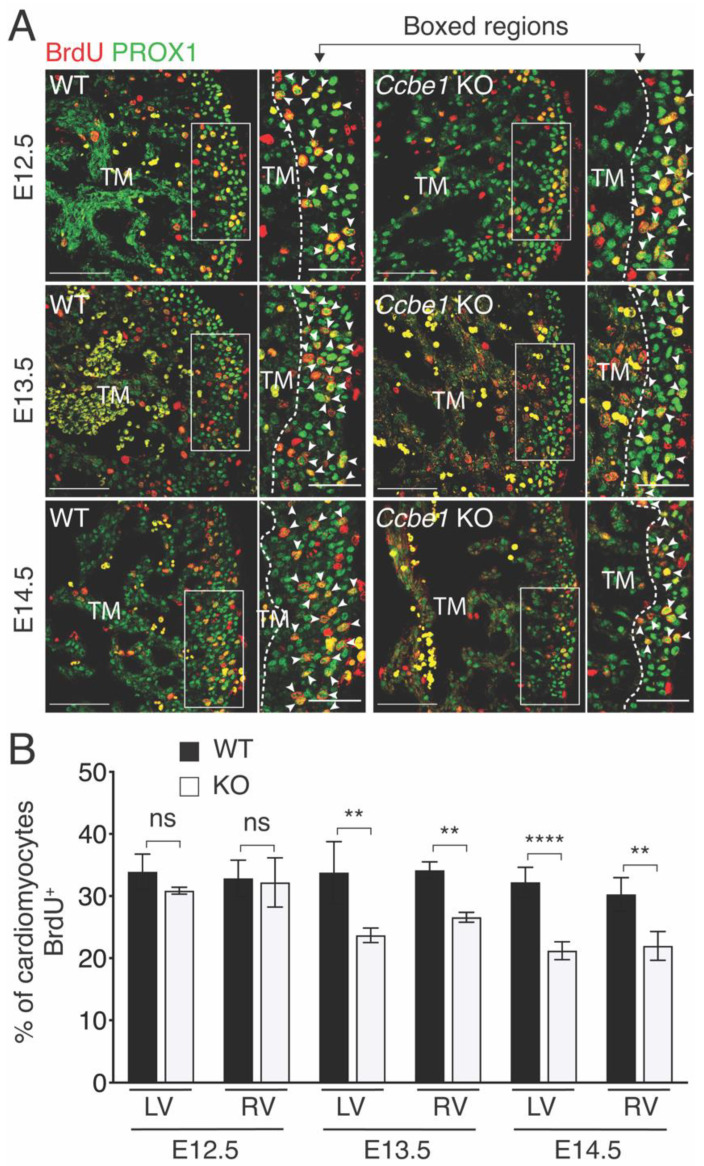
*Ccbe1^tm1Lex^* mice demonstrate less cardiomyocyte proliferation. (**A**) Tissue sections through E12.5, E13.5 and E14.5 wild-type and *Ccbe1* mutant hearts treated with BrdU and immunostained for BrdU and PROX1 to reveal proliferating cardiomyocytes. Arrowheads indicate BrdU^+^ cardiomyocytes. Images are representative of the following number of replicates: wild-type, *n* = 4; mutant, *n* = 4 at E12.5; wild-type, *n* = 5; mutant, *n* = 5 at E13.5; wild-type, *n* = 5; mutant, *n* = 5 at E14.5. (**B**) Quantification showed that cardiomyocyte proliferation in compact myocardium is reduced in *Ccbe1* mutant hearts at E.13.5 and E14.5. Dashed lines in (**A**) delimitate compact from trabecular myocardium. ns, nonsignificant; ** *p* < 0.01; **** *p* < 0.0001. Differences were considered statistically significant when *p* < 0.05. Error bars represent SDs. Scale bars = 100 μm. RV, right ventricle; LV, left ventricle; TM, trabecular myocardium.

Cardiomyocyte proliferation is required for compact myocardium formation starting around E13.0-E13.5. Accumulating evidence suggests that EPDC-cardiomyocyte crosstalk promotes this proliferation [21,22,23,24]. To further examine the nature of this defect, we assessed cardiomyocyte proliferation in the compact myocardium. BrdU incorporation assays using PROX1-specific antibodies as cardiomyocyte marker (Figure 2A) revealed that *Ccbe1* mutant hearts had a significantly decreased proliferation in the compact layer of both ventricles, as compared with wild-type hearts, at E13.5 and E14.5 (Figure 2B). No differences were observed at E12.5 (Figure 2B). Taken together, these results indicate that *Ccbe1^tm1Lex^* mice display abnormal ventricular development and growth.

### 2.2. CCBE1 Loss-of-Function Impairs Epicardial Function

**Figure 3:**
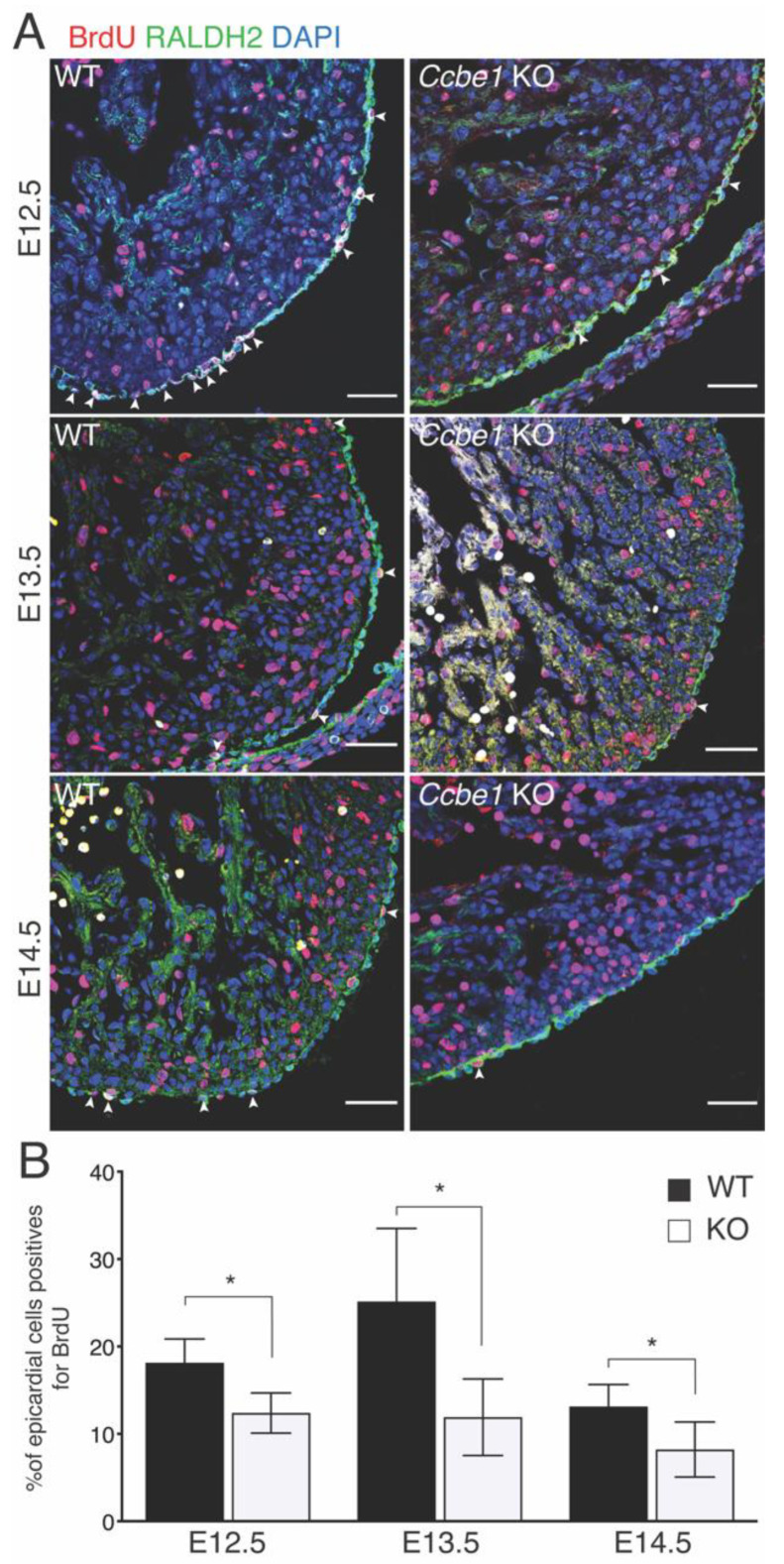
*Ccbe1^tm1Lex^* mice show less epicardial cell proliferation. (**A**) Transverse sections of E12.5 E13.5 and E14.5 wild-type and *Ccbe1* mutant hearts treated with BrdU, immunostained for BrdU, and RALDH2 to reveal proliferating epicardial cells. Arrowheads indicate BrdU^+^ epicardial cells. (**B**) Quantification shows that epicardial proliferation is reduced in *Ccbe1* mutant hearts. * *p* < 0.05. Differences were considered statistically significant when *p* < 0.05. Error bars represent SDs. Scale bars = 100 μm.

The epicardium provides an important cellular contribution to the developing heart. After migrating into the subepicardial space through the EMT, EPDCs differentiate into nonmyocyte cells, namely coronary endothelial cells, smooth muscle cells, fibroblasts, and pericytes that invade the intramyocardial wall [6,7,8]. The epicardium is also a source of secreted factors that play a critical role during myocardial compaction directing cardiomyocyte proliferation [21,22,23,24]. Since *Ccbe1* is expressed in the epicardium [18], we next evaluated how the lack of CCBE1 impacts epicardial development. Confocal analysis of E12.5, E13.5, and E14.5 heart sections using a RALDH2-specific antibody did not show an obvious defect in epicardial layer establishment (Figure 3A). We next asked whether KO of *Ccbe1* affects epicardial function. We performed a BrdU incorporation assay together with RALDH2-specific antibody to quantify epicardial cell proliferation. Figure 3A,B shows that *Ccbe1^tm1Lex^* hearts had a lower proliferation rate in epicardial cells than wild-type hearts at E12.5, E13.5 and E14.5 stages.

**Figure 4:**
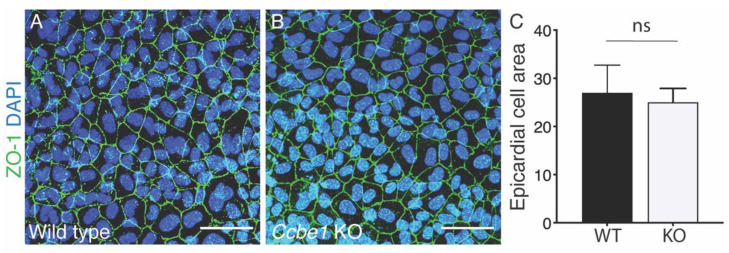
*Ccbe1* KO epicardial explants show normal size, morphology, and ZO-1 localization. (**A**,**B**) epicardial explants immunostained for ZO-1 antibody. Images are representative of the following number of replicates: wild-type, *n* = 12; mutant, *n* = 12. (**C**) Quantification shows no significant differences in epicardial cell areal. ns, nonsignificant. Scale bars = 50 μm.

We also analyzed the morphology of the epicardial cells using epicardial explants prepared from E11.5 hearts ventricles. Immunofluorescence staining with ZO-1 antibody to visualize the epicardial membrane showed no morphological difference of epicardial cells from *Ccbe1^tm1Lex^* explants as compared to wild-type (Figure 4A–C). In addition, CCBE1 loss-of-function did not affect ZO-1 expression or ZO-1 localization at the cell-cell junctions (Figure 4A,B). These results suggest that CCBE1 is not necessary for the establishment of the epicardium.

**Figure 5:**
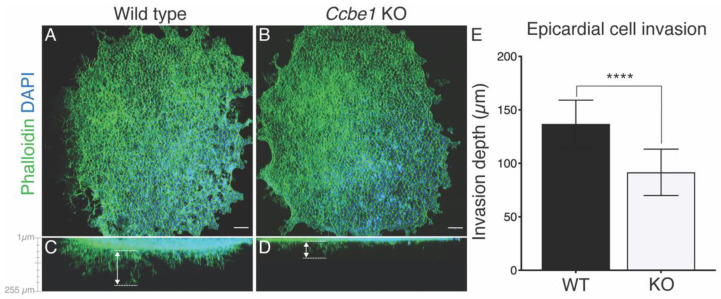
EPDC invasion is impairs in *Ccbe1* KO epicardial explants. (**A**,**B**) x/y plane of epicardial outgrowths from wild-type (**A**) and *Ccbe1* mutant (**B**) explants stained with phalloidin. (**C**,**D**) Confocal imaging of epicardial cell invasion along the z axis. Images are representative of the following number of replicates: wild-type, *n* = 11; mutant, *n* = 14. (**E**) Measurements along the z-axes showed a significant decrease in the depth of epicardial cell invasion in the *Ccbe1* KO explants compared with wild-type. **** *p* < 0.0001. Differences were considered statistically significant when *p* < 0.05. Error bars represent SDs. Scale bars = 100 μm.

Finally, we explored whether KO of *Ccbe1* also affects subsequent EPDC migration into the myocardium during mouse heart development. We employed a three-dimensional collagen gel invasion assay using E11.5 hearts ventricles. Analysis of these explants by confocal imaging using a Z-projection of the explant along the longitudinal axis allowed the measurement of cell invasion depth (μm) into the collagen gel matrix (Figure 5A–E). This analysis showed that epicardial outgrowth from *Ccbe1* KO explants covered less area and showed a significant reduction in invasion depth as compared with the wild-type (Figure 5A–E). Together, these observations suggest that lack of CCBE1 does not have an impact on epicardial cell morphology as epicardial cells align and organize properly, although the invading EPDCs are less invasive in *Ccbe1* KO epicardial explant, suggesting EMT impairment.

### 2.3. Loss of CCBE1 Impairs Proliferation of Nonmyocyte/Nonendothelial Cells

As mentioned, the myocardium depends on epicardial signals for its development. Moreover, the differentiation of EPDCs also depends on instructions from the adjacent myocardium, constituting a complex network of reciprocal epicardial–myocardial crosstalk essential for proper maturation of myocardium and EPDCs. In this sense, it has been shown that cardiac fibroblasts develop coincident with the growth of the compact layer [25]. Hence, we next examined the development of nonmyocyte/nonendothelial cells in *Ccbe1* KO hearts.

**Figure 6:**
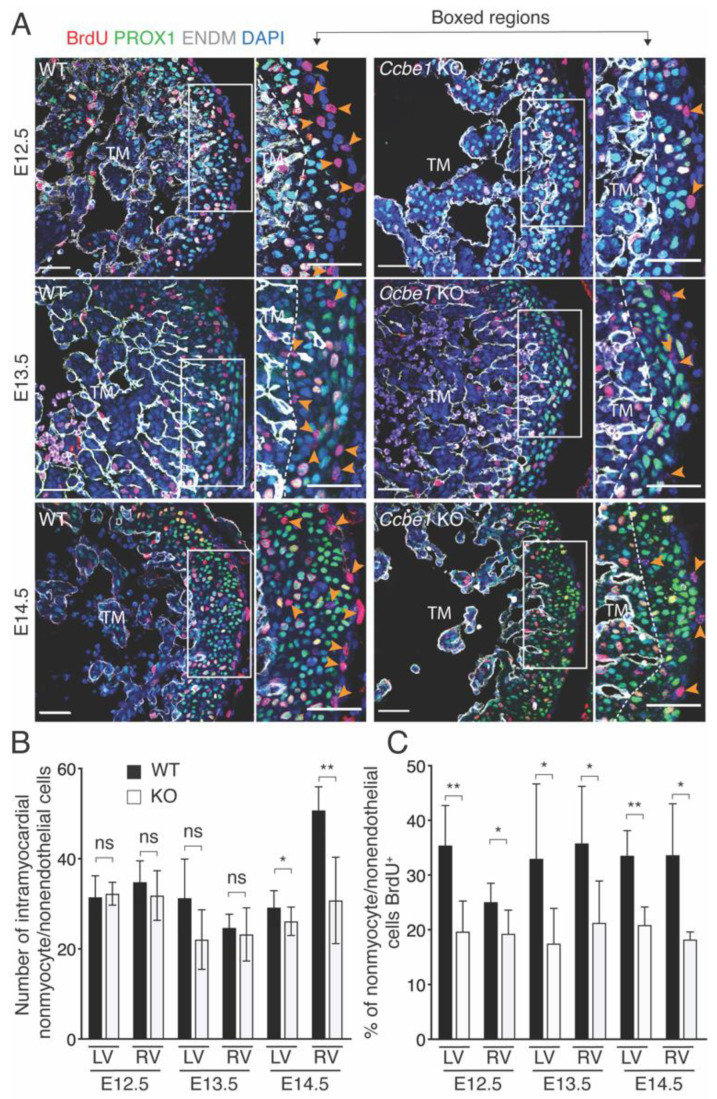
Nonmyocyte/nonendothelial cells are reduced and show less proliferation rate in *Ccbe1* KO hearts. (**A**) Tissue sections through E12.5, E13.5 and E14.5 wild-type and *Ccbe1* mutant hearts treated with BrdU and immunostained for BrdU, PROX1, and ENDM to reveal proliferating and nonproliferating nonmyocyte/nonendothelial cells. Orange arrowheads indicate proliferating (BrdU^+^) nonmyocyte/nonendothelial cells. Images are representative of the following number of replicates: wild-type, *n* = 5; mutant, *n* = 5 at E12.5; wild-type, *n* = 5; mutant, *n* = 6 at E13.5; wild-type, *n* = 5; mutant, *n* = 5 at E14.5. (**B**) Quantification showed reduced percentage of nonmyocyte/nonendothelial cells in right and left ventricles of *Ccbe1* mutant hearts at E14.5. (**C**) Quantification showed that nonmyocyte/nonendothelial cell proliferation in compact myocardium is reduced in right and left ventricles of *Ccbe1* mutant hearts at E12.5, E13.5 and E14.5. Dashed lines in (**A**) delimitate compact from trabecular myocardium. ns, nonsignificant; * *p* < 0.05; ** *p* < 0.01. Differences were considered statistically significant when *p* < 0.05. Error bars represent SDs. Scale bars = 100 μm. TM, trabecular myocardium.

To determine whether the lack of CCBE1 affected intramyocardial EPDCs, heart sections were immunostained with PROX1-specific antibody as cardiomyocyte marker and ENDM antibody as an endothelial marker. Nonmyocyte/nonendothelial cells were identified as negative for both PROX1 and ENDM and counted. Consistent with the defect in EPDC migration observed in epicardial explants from mutant hearts, the percentage of nonmyocyte/nonendothelial cells were less in *Ccbe1* KO hearts at E14.5 when compared to wild-type ones (Figure 6A,B).

We also assessed the proliferation capacity of nonmyocyte/nonendothelial cells in *Ccbe1^tm1Lex^* hearts. BrdU incorporation assays were performed using PROX1-specific antibody as cardiomyocyte marker and ENDM antibody as an endothelial marker. Nonmyocyte/nonendothelial cells were identified as negative for both PROX1 and ENDM. The ratio of proliferating nonmyocyte/nonendothelial cells (BrdU^+^) were significantly less in right and left ventricles of *Ccbe1^tm1Lex^* hearts from E12.5 to E14.5 as compared to wild-type hearts (Figure 6A,C). These results indicate that CCBE1 is required for proper migration of EPDC into the myocardium, and also for nonmyocyte/nonendothelial cell proliferation.

### 2.4. Lack of CCBE1 Affect Epicardial EMT Signaling

**Figure 7:**
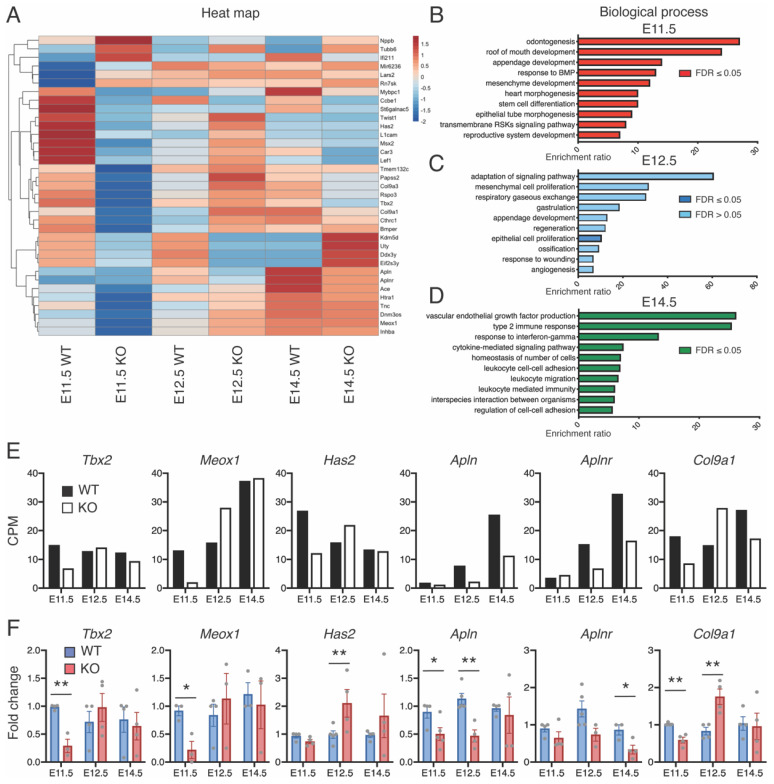
*Ccbe1* KO hearts exhibit deregulation of expression of EMT- and coronary development-related genes. (**A**) Heatmap of differentially regulated genes in *Ccbe1^tm1Lex^* ventricles at E11.5 (*n* = 3), E12.5 (*n* = 3), and E14.5 (*n* = 3) as compared to wild-type hearts (*n* = 3, respectively). (**B**–**D**) GO functional enrichment of DEGs in *Ccbe1* KO ventricles at E11.5 (**B**), E12.5 (**C**), and E13.5 (**D**). (**E**) Gene expression for differentially expressed genes. (**F**) qRT-PCR validation. * *p* < 0.05; ** *p* < 0.01. Differences were considered statistically significant when * *p* < 0.05. Error bars represent SDs. CPM, counts per million reads mapped.

To identify molecular pathways responsible for altered phenotype and function of *Ccbe1* KO hearts, we performed a genome-wide transcriptomic analysis of E11.5, E12.5 and E14.5 ventricles from *Ccbe1^tm1Lex^* and wild-type mice by RNA-seq. RNA-seq data was analyzed to identify differentially expressed genes (DEGs) (Figure 7A) and the corresponding dysregulated biological pathways (Figure 7B–D). The differences in the expression patterns of *Ccbe1* KO hearts samples and their wild-type controls are evident in the heat map representation (Figure 7A). Most downregulated genes in E11.5 *Ccbe1* KO hearts belonged to the GO (biological process) classes response to BMP (*Htra1*, *Msx2*, *Lef1,* and *Bmper*), mesenchyme development (*Tbx2*, *Meox1*, *Msx2*, *Has2*, *Lef1,* and *Twist1*), heart morphogenesis and epithelial tube morphogenesis (*Tbx2*, *Msx2*, *Lef1*, *Tnc*, *Twist1*, and *Cthrc1*), among others (Figure 7B). Whereas genes down and upregulated in E12.5 *Ccbe1* KO hearts belonged to epithelial cell proliferation (*Sfrp2*, *Osr1*, *Eppk1*, *Apln*, and *Aplnr*) (Figure 7C). Finally, most deregulated genes in E14.5 *Ccbe1* KO hearts belonged to vascular endothelial growth factor production (*Ccl2* and *Ccr2*) and regulation of cell-cell adhesion (*Ptpn6*, *Arg1*, *Cd74*, *Il1rn*, *Gpnmb*, *Ccl2*, and *Ccr2*) (Figure 7D). Next, we performed an experimental validation using qRT-PCR selecting genes such as *Tbx2*, *Meox1*, *Has2*, *Col9a1*, *Apln*, and *Aplnr* as these genes are known to play a role in epicardial development (*Tbx2*, *Meox1*, and *Has2*) [26,27,28] and coronary vasculature development (*Apln* and *Aplnr*) [29] processes affected in *Ccbe1* KO hearts [18]. Col9a1 was selected as cardiac extracellular matrix component [30]. For qRT-PCR confirmation, all six genes showed a strong correlation between RNA-seq and qRT-PCR (Figure 7E,F). These results suggest that CCBE1 loss-of-function impairs signaling pathways that are involved in EMT and coronary vasculature development.

## 3. Discussion

Our study shows that CCBE1 deficiency alters epicardial function. The reduced epicardial cell proliferation and migration, together downregulation of EMT-related genes, suggest defects in the proper function of the epicardium. Consequently, the observed impairment of epicardial function had an impact on the myocardial growth as *Ccbe1^tm1Lex^* mice displayed reduced ventricular myocardium thickness.

Recently, the role of the epicardium in heart development has been subjected to extensively studies [Reviewed in 9]. In addition to directly differentiating into various cell lineages through epicardial EMT, the epicardium also supports cardiomyocyte proliferation and embryonic heart growth by providing a cocktail of secreted mitogens that become possible EPDC-cardiomyocyte crosstalk [9]. Accordingly, ablation of the epicardium or genes expressed in the epicardium disrupts myocardial growth, coronary vessel development, and the formation of cardiac interstitial cells [31,32,33,34,35,36,37,38,39,40,41,42,43,44]. We previously reported that *Ccbe1* is expressed in the epicardium of the developing heart, and mice lacking CCBE1 have diminished coronary development [18]. Here, we performed a deeper analysis of the heart phenotype in histological sections from *Ccbe1^tm1Lex^* hearts, and we observed that *Ccbe1* mutant mice displayed thinner right and left ventricle walls, as well as increased trabeculation, from E12.5 to E.14.5. No analysis could be performed at later stages because mutants die shortly after E14.5 [15]. Concordantly, the mutant hearts showed a decreased cardiomyocyte proliferation in the compact myocardium, which could be a pivotal factor leading to the thinner ventricular walls observed. Interestingly, abolishing proliferation in the compact myocardium leads to a hyper-trabeculation phenotype [45]. More recently, it was described that the suppression of *Prdm16* gene, which promotes the expression of genes required for compact myocardium growth, leads to an upregulation of genes involved in trabecular growth [46]. Thus, the large trabecular myocardium displayed by *Ccbe1* mutant hearts in both ventricles could be explained by an impairment in this compact-trabecular interplay.

The phenotype observed in *Ccbe1* KO mice is in line with those observed upon ablation of the epicardium or genes expressed in the epicardium [31,32,33,34,35,36,37,38,39,40,41,42,43,44], further suggesting that epicardial CCBE1 is necessary for epicardial function during development.

Following epicardium development, once it is fully established, the epicardial cells undergo EMT, promoting proliferation, migration, and differentiation of EPDCs in order to support the coronary vasculature and ventricular development. During this process, subsets of cells in the epicardium proliferate to form more epicardial cells or EPDCs that will later be differentiated into SMCs, endothelial cells, and fibroblast [47]. We then analyzed whether epicardial phenotype and function were impaired in *Ccbe1* mutants. Although mutants had no obvious alterations in the establishment of the epicardial layers, we observed a reduced epicardial cell proliferation in *Ccbe1* mutant hearts. Moreover, in the epicardial explants, the cobblestone-like morphology of the cells (epithelial) in the epicardial monolayer was identical in wild-type and *Ccbe1* KO explants, and no enlarged cells (mesenchymal) were observed. However, we observed impaired collagen gel invasion of EPDCs derived from explants with *Ccbe1* KO, suggesting again disrupted EMT and EPDC migration. Altogether, this is consistent with the impaired EMT observed after deletion of genes expressed in the epicardium, including *Wt1*, *ß-catenin*, *Tcf21*, *Mrtfa* and *Mrtfb*, *Nfatc1*, and *Prmt1* [31,35,38,42,44,48,49]. Coronary endothelial cells have been suggested to also stimulate cardiomyocyte proliferation supporting myocardial expansion [50]. Therefore, the lack of coronary endothelium in *Ccbe1* KO hearts [18], could also account for their reduced ventricular compact myocardium thickness. However, this coronary endothelium-cardiomyocyte crosstalk was only proved in an in vitro model, and no in vivo evidence has been reported [50]. These observations suggest that the defect in the compact myocardial observed in our mutant is most likely due to epicardial function disruption.

After epicardial EMT, EPDCs migrate further into the myocardium and differentiate into nonmyocyte cells [6,7,8]. The complex network of reciprocal epicardial–myocardial crosstalk plays a critical role in maturation of myocardium. We have shown a reduced cardiomyocyte proliferation rate in the compact myocardium, accompanied by an impairment of epicardial function in *Ccbe1* mutant hearts. Consistently, the number of nonmyocyte/nonendothelial cells present intramyocardially was smaller in E14.5 *Ccbe1* KO hearts when compared to wild-types. In addition, these cell populations also showed less proliferation rate in *Ccbe1* mutant hearts in all the analyzed stages.

The EMT is induced by transcription factors, including Snail Family Transcriptional Repressors 1 and 2 (SNAI1/2), zinc finger E-box binding family members 1 and 2 (ZEB1/2), and twist-related protein 1 (TWIST1) [51] that, simultaneously, repress epithelial genes, such as E-Cadherin, responsible of maintaining cell-cell adhesion and adherent junctions, and activate the expression of mesenchymal genes. Curiously, our RNA-seq analysis of *Ccbe1* mutant ventricles and subsequent GO analysis revealed downregulation of genes involved in mesenchyme development (*Tbx2*, *Meox1*, *Msx2*, *Has2*, *Lef1*, and *Twist1*) and response to BMP, a signaling pathway known to regulate epicardial cell invasion during development [52,53]. Furthermore, most of those genes, including *Twist1*, *Has2*, *Bmper*, *Lef1*, *Htra1*, *Tbx2*, and *Msx2* have been identified as EMT related genes [26,38,54,55,56,57,58,59]. These data together with our observation in vitro and in vivo indicate that the epicardial EMT is impaired in *Ccbe1* mutant hearts.

The ECM is the protein scaffold that supports cell attachment and biochemical and biomechanical signaling cues to surrounding cells [60]. EMT-inducer transcription factor activates mesenchymal genes necessary for ECM production and cell migration [9,57]. In addition, ECM components have been described to play an essential role during EMT [61]. Surprisingly, the RNA-seq-based GO analysis also revealed downregulation of ECM genes, including *Col9a1*, *Col9a3*, *Cthrc1*, and *Tnc*, supporting the involvement of CCBE1 in the epicardial EMT.

Our results show that decreased epicardial cell proliferation and EPDC invasion are associated with reduced ventricular myocardium thickness and are, at the same time, supported by several other gene knockouts that show similarities [31,32,33,34,35,36,37,38,39,40,41,42,43,44]. Epicardial derivatives are a potential source of cells for the development of therapies to repair the damaged heart [10,62,63,64]. A mounting amount of evidence demonstrates that pathways that regulate epicardial development are reinitiated during heart regeneration. Hence, understanding the factors that regulate epicardial cell proliferation and invasion during development may become an opportunity to target the epicardium to modulate the response to injury in adults. Besides its potential as a potential target for coronary neovascularization [18], our data also suggest CCBE1 as a novel potential therapeutic target to promote cardiac repair through modulating epicardial cell proliferation and invasion.

Overall, our findings suggest that CCBE1 plays a pivotal role during epicardial development by regulating epicardial cell proliferation and EPDC migration into the myocardium. Moreover, the lack of CCBE1 led to the downregulation of EMT-related genes indicating a role in modulating the EMT process. Hence, based on our observations, we propose that the reduced ventricular myocardium thickness phenotype of *Ccbe1^tm1Lex^* mutant hearts is due to an impairment in epicardial function.

## 4. Materials and Methods

### 4.1. Mice and Ethics Statement

The *Ccbe1* mutant mouse (*Ccbe1^tm1Lex^*; Lexicon Pharmaceuticals Inc. The Woodlands, TX, USA) is a conventional KO mouse in pure C57Bl/6 background in which the first two exons of the gene have been targeted and replaced by cDNA-encoding β-galactosidase [65]. Heterozygote *Ccbe1^tm1Lex^* are viable. Homozygote *Ccbe1^tm1Lex^* were obtained upon crossing male and female heterozygote *Ccbe1^tm1Lex^*, and referred hereafter as *Ccbe1* KO. Euthanasia was performed using carbon dioxide in gradual fill followed by cervical dislocation. All animal experiments were performed in accordance with the European Union (EU) guidelines for animal research and welfare, and in compliance with the Portuguese law and approved by the Consultative Commission of the Veterinary Agency from Portuguese Ministry of Agriculture (Directive 2010/63/EU of the European Parliament).

### 4.2. Histology and Immunofluorescence

For timed pregnancies, vaginal plugs were checked in the morning after mating, noon was taken as E0.5. Embryos of developmental stages between E11.5 and E14.5 were isolated for analysis. They were dissected in PBS and fixed over-night with 4% PFA, dehydrated and stored in 100% (*v*/*v*) ethanol (Merck, KGaA, Darmstadt, Germany) at 4 °C until paraffin embedding and transverse sectioning with a microtome (Microm HM200, 7 um). Immunofluorescence was performed on microscope slides. Citrate buffer pH6 (Vector Laboratories, Newark, CA, USA) was used for heat-induced antigen retrieval, for a period of 20 min. Microscope slides were washed in PBT for 15 min at room temperature and incubated in blocking solution for 2 h at room temperature. Primary antibodies were diluted in blocking solution and incubated over-night at 4 °C followed by PBT washes for 30 min. Secondary antibodies diluted in blocking solution were incubated 2 h at room temperature and washed again in PBT.

For ENDM and RALDH2 antibodies, signal was amplified using biotilinylated α-Rat and α-Rabbit antibody, respectively (Vectastain ABC HRP Kit; Rat IgG, PK-4004, and Rabbit IgG, PK-4001), following the manufacture instructions and a Streptavidin FITC Conjugated (Sigma-Aldrich, St. Louis, MO, USA), E2761). Sections were mounted with VECTASHIELD^®^ Mounting Media with DAPI (Vector Laboratories; Burlingame, CA, USA) and imaged using a ZEISS LSM-710 microscope (ZEISS, Oberkochen, Germany). Image processing used either ZEN (black edition, ZEISS) or ImageJ (NIH).

Antibodies were BrdU (Roche, Mannheim, Germany, 1117037600; 1:100), RALDH2 (Abcam, Cambridge, UK, ab7567; 1:200), ZO-1 (LSBio, Seattle, WA, USA, LS-C50462-50; 1:200), Prox1 (R&D Systems, Minneapolis, MN, USA, AF2727; 1:200), and ENDM (Santa Cruz Biotechnology, CA, USA, SC65495, 1:300). Secondary antibodies were Alexa Fluor conjugates (488, 594, 633, Life Technologies, Carlsbad, CA, USA; 1:250).

### 4.3. Quantification of Compact Myocardium Thickness and Trabecular Length

To visualize the structure of ventricles, immunostaining was performed on paraffin sections with ENDM antibody for endocardial cells. ImageJ software was used to measure the thickness of the compact myocardium and the length of trabecular myocardium in tissue sections from equivalent coronal planes of the heart at the atrioventricular septum level. For each parameter, three measurements were taken along the lateral sides of each ventricle, and averaged individually, in three serial sections.

### 4.4. BrdU Proliferation Assays

In vivo labeling of proliferating cells was performed by intraperitoneal injection of 5-Bromo-2-deoxyuridine (BrdU, Roche, Mannheim, Germany) at 100 mg/kg of body weight, using a sterile solution of 10 mg/mL BrdU dissolved in PBS. Pregnant females were injected 2 h before embryo collection. Proliferating cardiomyocytes and nonmyocyte/nonendothelial cells were calculated from paraffin tissue sections as the percentage of PROX1-positive cardiomyocytes or PROX1 and ENDM negatives cells, respectively, labeled BrdU in a 20,000 µm^2^ field of view in three serial sections.

### 4.5. Epicardial Explant Culture

Collagen gels (1.2%) were prepared as previously reported by Runyan & Markwald, 1983 [66]. Minor modifications were performed. In brief, type I rat tail collagen (BD Biosciences, San José, CA, USA) was mixed with 10XM199 (Gibco, Palo Alto, CA, USA) medium, sterile water, and 2.2% sodium bicarbonate. Gels were allowed to solidify in a four-well chamber (Nalge Nunc International, Rochester, NY, USA) for 30 min at 37 °C and washed with Dulbecco’s modified Eagle’s medium (DMEM) (Gibco, Palo Alto, CA, USA). Gels were then incubated O/N at 37 °C in DMEM supplemented with 10% fetal bovine serum (FBS), insulin transferrin selenium-X (ITS), and penicillin/streptomycin (P/S).

E11.5 hearts were collected, and the atrial and outflow tract were removed. The hearts were placed on the gels with the ventral face down to maximize the contact area of the epicardial cells and to prevent the contact of the exposed endocardium with the collagen gel. Hearts were then cultured for 48 h allowing them to attach and cells grow out on the gel with DMEM containing 10% FBS, ITS, and P/S. Next, the hearts were removed, and the explants were cultured for an additional 2 days. Gels were washed with PBS twice, fixed in 4% PFA and stained with phalloidin. Nuclei were counterstained with DAPI.

To measure cell invasion depth, z-stacks were taken through the thickness of each collagen gel. Using most profound 20–30 cells, deep were measured relative to the surface of the explant using ImageJ (NIH).

### 4.6. RNA Isolation and qRT-PCR

Hearts were isolated at E11.5, E12.5, and E14.5 stages. Atria and valves were removed, and ventricles were pooled (*n* = 3) and stored at −80 °C until used. Total RNA was extracted and purified by using Trizol reactive (Invitrogen, Carlsbad, CA, USA) according to the manufacturer’s instructions.

For the reverse transcription reaction (first-strand cDNA synthesis), RevertAid Reverse Transcriptase, oligo-dT primer, RiboLock RNAse Inhibitor, and dNTPs (Thermo Fisher Scientific, Carlsbad, CA, USA) were used following the manufacturer’s protocol and using 10 μg of RNA per sample. cDNA samples were diluted 1:5 with nuclease-free water and frozen until used.

qPCR was performed on ABI QuantStudio 5 Real-Time PCR System (Thermo Fisher Scientific, Carlsbad, CA, USA) using Power SYBR Green PCR Master Mix (Applied Biosystems, Foster City, CA, USA). The Cycle threshold (Ct) was determined using Design and Analysis software (Thermo Fisher App). The results were analyzed as described in Livak & Schmittgen 2001, using the 2^−ΔΔCT^ method for relative gene expression analysis [67]. The gene expression data were normalized using two housekeeping genes, *Gapdh* and *Pgk1*, and represented relative to a control sample (set at 1).

### 4.7. RNA-Seq Analysis and Bioinformatics

Quality and integrity of total RNA were controlled on the Agilent Technologies 2100 Bioanalyzer. Libraries were generated using Stranded mRNA Library Prep Kit, and pair-end libraries were sequenced on an Illumina PE150 Platform with an output of ~40M reads per sample. The reads quality was assessed using FastQC software and mapping of reads was performed applying the STAR (Version 2.7.5c) aligner using murine genome from GENCODE Release M25 as reference [68] In particular, the primary genome sequence assembly GRCm38 together with corresponding annotations was used. The read counts per gene were obtained executing the featureCount function of the Bioconductor package Rsubread (Version 2.2.6) [69]. The transcripts per million (TPM) were then calculated as gene expression measure. Differential gene expression analysis was performed using Bioconductor edgeR package (Version 3.30.3) with a biological coefficient of variation of 0.2 [70] (McCarthy et al., 2012) and *p*-value correction for multiple testing was performed using the Benjamini Hochberg (FDR) method. Finally, mappings of Ensembl IDs to gene symbols were extracted from Bioconductor package org.Mm.eg.db for murine genome annotation (Version 3.11.4). As functional enrichment analysis for KEGG pathway categories, Over-Representation Analysis of differentially expressed genes was conducted using WebGestalt [69].

### 4.8. Statistics

For statistical analyses of datasets, unpaired Student’s t-tests were used. Significance levels or *p* values are stated in each corresponding figure legend. *p* < 0.05 was considered statistically significant. All the statistical analyses were performed using GraphPad Prism 9.0 software (San Diego, CA, USA).

## Data Availability

The RNA-seq data presented in this study are openly available in the EMBL-EBI ArrayExpress under accession number E-MTAB-12217.

## References

[1] Sedmera D., Pexieder T., Vuillemin M., Thompson R.P., Anderson R.H. (2000). Developmental Patterning of the Myocardium. Anat. Rec..

[2] Choquet C., Kelly R.G., Miquerol L. (2019). Defects in Trabecular Development Contribute to Left Ventricular Noncompaction. Pediatr. Cardiol..

[3] Sedmera D., Thomas P.S. (1996). Trabeculation in the Embryonic Heart. Bioessays.

[4] Winter E.M., Gittenberger-de Groot A.C. (2007). Epicardium-Derived Cells in Cardiogenesis and Cardiac Regeneration. Cell. Mol. Life Sci..

[5] Gittenberger-de Groot A.C., Winter E.M., Bartelings M.M., Goumans M.J., DeRuiter M.C., Poelmann R.E. (2012). The Arterial and Cardiac Epicardium in Development, Disease and Repair. Differentiation.

[6] Poelmann R.E., Gittenberger-de Groot A.C., Mentink M.M., Bökenkamp R., Hogers B. (1993). Development of the Cardiac Coronary Vascular Endothelium, Studied with Antiendothelial Antibodies, in Chicken-Quail Chimeras. Circ. Res..

[7] Mikawa T., Gourdie R.G. (1996). Pericardial Mesoderm Generates a Population of Coronary Smooth Muscle Cells Migrating into the Heart along with Ingrowth of the Epicardial Organ. Dev. Biol..

[8] Dettman R.W., Denetclaw W., Ordahl C.P., Bristow J. (1998). Common Epicardial Origin of Coronary Vascular Smooth Muscle, Perivascular Fibroblasts, and Intermyocardial Fibroblasts in the Avian Heart. Dev. Biol..

[9] Quijada P., Trembley M.A., Small E.M. (2020). The Role of the Epicardium During Heart Development and Repair. Circ. Res..

[10] Lepilina A., Coon A.N., Kikuchi K., Holdway J.E., Roberts R.W., Burns C.G., Poss K.D. (2006). A Dynamic Epicardial Injury Response Supports Progenitor Cell Activity during Zebrafish Heart Regeneration. Cell.

[11] Russell J.L., Goetsch S.C., Gaiano N.R., Hill J.A., Olson E.N., Schneider J.W. (2011). A Dynamic Notch Injury Response Activates Epicardium and Contributes to Fibrosis Repair. Circ. Res..

[12] Zhou B., Pu W.T. (2011). Epicardial Epithelial-to-Mesenchymal Transition in Injured Heart. J. Cell. Mol. Med..

[13] Van Wijk B., Gunst Q.D., Moorman A.F.M., van den Hoff M.J.B. (2012). Cardiac Regeneration from Activated Epicardium. PLoS ONE.

[14] Wang J., Cao J., Dickson A.L., Poss K.D. (2015). Epicardial Regeneration Is Guided by Cardiac Outflow Tract and Hedgehog Signalling. Nature.

[15] Bos F.L., Caunt M., Peterson-Maduro J., Planas-Paz L., Kowalski J., Karpanen T., van Impel A., Tong R., Ernst J.A., Korving J. (2011). CCBE1 Is Essential for Mammalian Lymphatic Vascular Development and Enhances the Lymphangiogenic Effect of Vascular Endothelial Growth Factor-C in Vivo. Circ. Res..

[16] Le Guen L., Karpanen T., Schulte D., Harris N.C., Koltowska K., Roukens G., Bower N.I., van Impel A., Stacker S.A., Achen M.G. (2014). Ccbe1 Regulates Vegfc-Mediated Induction of Vegfr3 Signaling during Embryonic Lymphangiogenesis. Development.

[17] Chen H.I., Sharma B., Akerberg B.N., Numi H.J., Kivelä R., Saharinen P., Aghajanian H., McKay A.S., Bogard P.E., Chang A.H. (2014). The Sinus Venosus Contributes to Coronary Vasculature through VEGFC-Stimulated Angiogenesis. Development.

[18] Bonet F., Pereira P.N.G., Bover O., Marques S., Inácio J.M., Belo J.A. (2018). CCBE1 Is Required for Coronary Vessel Development and Proper Coronary Artery Stem Formation in the Mouse Heart. Dev. Dyn..

[19] Jakus Z., Gleghorn J.P., Enis D.R., Sen A., Chia S., Liu X., Rawnsley D.R., Yang Y., Hess P.R., Zou Z. (2014). Lymphatic Function Is Required Prenatally for Lung Inflation at Birth. J. Exp. Med..

[20] Burger N.B., Bekker M.N., Kok E., De Groot C.J.M., Martin J.F., Shou W., Scambler P.J., Lee Y., Christoffels V.M., Haak M.C. (2015). Increased Nuchal Translucency Origins from Abnormal Lymphatic Development and Is Independent of the Presence of a Cardiac Defect. Prenat. Diagn..

[21] Chen T.H.P., Chang T.-C., Kang J.-O., Choudhary B., Makita T., Tran C.M., Burch J.B.E., Eid H., Sucov H.M. (2002). Epicardial Induction of Fetal Cardiomyocyte Proliferation via a Retinoic Acid-Inducible Trophic Factor. Dev. Biol..

[22] Lavine K.J., Yu K., White A.C., Zhang X., Smith C., Partanen J., Ornitz D.M. (2005). Endocardial and Epicardial Derived FGF Signals Regulate Myocardial Proliferation and Differentiation in Vivo. Dev. Cell.

[23] Li P., Cavallero S., Gu Y., Chen T.H.P., Hughes J., Hassan A.B., Brüning J.C., Pashmforoush M., Sucov H.M. (2011). IGF Signaling Directs Ventricular Cardiomyocyte Proliferation during Embryonic Heart Development. Development.

[24] Shen H., Cavallero S., Estrada K.D., Sandovici I., Kumar S.R., Makita T., Lien C.-L., Constancia M., Sucov H.M. (2015). Extracardiac Control of Embryonic Cardiomyocyte Proliferation and Ventricular Wall Expansion. Cardiovasc. Res..

[25] Ieda M., Tsuchihashi T., Ivey K.N., Ross R.S., Hong T.-T., Shaw R.M., Srivastava D. (2009). Cardiac Fibroblasts Regulate Myocardial Proliferation through Beta1 Integrin Signaling. Dev. Cell.

[26] Craig E.A., Parker P., Austin A.F., Barnett J.V., Camenisch T.D. (2010). Involvement of the MEKK1 Signaling Pathway in the Regulation of Epicardial Cell Behavior by Hyaluronan. Cell. Signal..

[27] Greulich F., Rudat C., Farin H.F., Christoffels V.M., Kispert A. (2016). Lack of Genetic Interaction between Tbx18 and Tbx2/Tbx20 in Mouse Epicardial Development. PLoS ONE.

[28] Velecela V., Torres-Cano A., García-Melero A., Ramiro-Pareta M., Müller-Sánchez C., Segarra-Mondejar M., Chau Y.-Y., Campos-Bonilla B., Reina M., Soriano F.X. (2019). Epicardial Cell Shape and Maturation Are Regulated by Wt1 via Transcriptional Control of Bmp4. Development.

[29] Tian X., Hu T., Zhang H., He L., Huang X., Liu Q., Yu W., He L., Yang Z., Zhang Z. (2013). Subepicardial Endothelial Cells Invade the Embryonic Ventricle Wall to Form Coronary Arteries. Cell Res..

[30] Gil-Cayuela C., Roselló-LLetí E., Ortega A., Tarazón E., Triviño J.C., Martínez-Dolz L., González-Juanatey J.R., Lago F., Portolés M., Rivera M. (2016). New Altered Non-Fibrillar Collagens in Human Dilated Cardiomyopathy: Role in the Remodeling Process. PLoS ONE.

[31] Moore A.W., McInnes L., Kreidberg J., Hastie N.D., Schedl A. (1999). YAC Complementation Shows a Requirement for Wt1 in the Development of Epicardium, Adrenal Gland and throughout Nephrogenesis. Development.

[32] Merki E., Zamora M., Raya A., Kawakami Y., Wang J., Zhang X., Burch J., Kubalak S.W., Kaliman P., Izpisua Belmonte J.C. (2005). Epicardial Retinoid X Receptor Alpha Is Required for Myocardial Growth and Coronary Artery Formation. Proc. Natl. Acad. Sci. USA.

[33] Lavine K.J., White A.C., Park C., Smith C.S., Choi K., Long F., Hui C., Ornitz D.M. (2006). Fibroblast Growth Factor Signals Regulate a Wave of Hedgehog Activation That Is Essential for Coronary Vascular Development. Genes Dev..

[34] Compton L.A., Potash D.A., Brown C.B., Barnett J.V. (2007). Coronary Vessel Development Is Dependent on the Type III Transforming Growth Factor Beta Receptor. Circ. Res..

[35] Zamora M., Männer J., Ruiz-Lozano P. (2007). Epicardium-Derived Progenitor Cells Require Beta-Catenin for Coronary Artery Formation. Proc. Natl. Acad. Sci. USA.

[36] Sridurongrit S., Larsson J., Schwartz R., Ruiz-Lozano P., Kaartinen V. (2008). Signaling via the Tgf-Beta Type I Receptor Alk5 in Heart Development. Dev. Biol..

[37] Mellgren A.M., Smith C.L., Olsen G.S., Eskiocak B., Zhou B., Kazi M.N., Ruiz F.R., Pu W.T., Tallquist M.D. (2008). Platelet-Derived Growth Factor Receptor Beta Signaling Is Required for Efficient Epicardial Cell Migration and Development of Two Distinct Coronary Vascular Smooth Muscle Cell Populations. Circ. Res..

[38] Von Gise A., Zhou B., Honor L.B., Ma Q., Petryk A., Pu W.T. (2011). WT1 Regulates Epicardial Epithelial to Mesenchymal Transition through β-Catenin and Retinoic Acid Signaling Pathways. Dev. Biol..

[39] Brade T., Kumar S., Cunningham T.J., Chatzi C., Zhao X., Cavallero S., Li P., Sucov H.M., Ruiz-Lozano P., Duester G. (2011). Retinoic Acid Stimulates Myocardial Expansion by Induction of Hepatic Erythropoietin Which Activates Epicardial Igf2. Development.

[40] Vega-Hernández M., Kovacs A., De Langhe S., Ornitz D.M. (2011). FGF10/FGFR2b Signaling Is Essential for Cardiac Fibroblast Development and Growth of the Myocardium. Development.

[41] Cavallero S., Shen H., Yi C., Lien C.-L., Kumar S.R., Sucov H.M. (2015). CXCL12 Signaling Is Essential for Maturation of the Ventricular Coronary Endothelial Plexus and Establishment of Functional Coronary Circulation. Dev. Cell.

[42] Trembley M.A., Velasquez L.S., de Mesy Bentley K.L., Small E.M. (2015). Myocardin-Related Transcription Factors Control the Motility of Epicardium-Derived Cells and the Maturation of Coronary Vessels. Development.

[43] Xiao Y., Hill M.C., Zhang M., Martin T.J., Morikawa Y., Wang S., Moise A.R., Wythe J.D., Martin J.F. (2018). Hippo Signaling Plays an Essential Role in Cell State Transitions during Cardiac Fibroblast Development. Dev. Cell.

[44] Jackson-Weaver O., Ungvijanpunya N., Yuan Y., Qian J., Gou Y., Wu J., Shen H., Chen Y., Li M., Richard S. (2020). PRMT1-P53 Pathway Controls Epicardial EMT and Invasion. Cell Rep..

[45] Tian X., Li Y., He L., Zhang H., Huang X., Liu Q., Pu W., Zhang L., Li Y., Zhao H. (2017). Identification of a Hybrid Myocardial Zone in the Mammalian Heart after Birth. Nat. Commun..

[46] Wu T., Liang Z., Zhang Z., Liu C., Zhang L., Gu Y., Peterson K.L., Evans S.M., Fu X.-D., Chen J. (2022). PRDM16 Is a Compact Myocardium-Enriched Transcription Factor Required to Maintain Compact Myocardial Cardiomyocyte Identity in Left Ventricle. Circulation.

[47] Von Gise A., Pu W.T. (2012). Endocardial and Epicardial Epithelial to Mesenchymal Transitions in Heart Development and Disease. Circ. Res..

[48] Combs M.D., Braitsch C.M., Lange A.W., James J.F., Yutzey K.E. (2011). NFATC1 Promotes Epicardium-Derived Cell Invasion into Myocardium. Development.

[49] Acharya A., Baek S.T., Huang G., Eskiocak B., Goetsch S., Sung C.Y., Banfi S., Sauer M.F., Olsen G.S., Duffield J.S. (2012). The BHLH Transcription Factor Tcf21 Is Required for Lineage-Specific EMT of Cardiac Fibroblast Progenitors. Development.

[50] Rhee S., Chung J.I., King D.A., D’amato G., Paik D.T., Duan A., Chang A., Nagelberg D., Sharma B., Jeong Y. (2018). Endothelial Deletion of Ino80 Disrupts Coronary Angiogenesis and Causes Congenital Heart Disease. Nat. Commun..

[51] Nieto M.A., Huang R.Y.-J., Jackson R.A., Thiery J.P. (2016). EMT: 2016. Cell.

[52] Sánchez N.S., Barnett J.V. (2012). TGFβ and BMP-2 Regulate Epicardial Cell Invasion via TGFβR3 Activation of the Par6/Smurf1/RhoA Pathway. Cell. Signal..

[53] Hill C.R., Sanchez N.S., Love J.D., Arrieta J.A., Hong C.C., Brown C.B., Austin A.F., Barnett J.V. (2012). BMP2 Signals Loss of Epithelial Character in Epicardial Cells but Requires the Type III TGFβ Receptor to Promote Invasion. Cell. Signal..

[54] Chen Y.-H., Ishii M., Sucov H.M., Maxson R.E. (2008). Msx1 and Msx2 Are Required for Endothelial-Mesenchymal Transformation of the Atrioventricular Cushions and Patterning of the Atrioventricular Myocardium. BMC Dev. Biol..

[55] Craig E.A., Austin A.F., Vaillancourt R.R., Barnett J.V., Camenisch T.D. (2010). TGFβ2-Mediated Production of Hyaluronan Is Important for the Induction of Epicardial Cell Differentiation and Invasion. Exp. Cell Res..

[56] Wang B., Lindley L.E., Fernandez-Vega V., Rieger M.E., Sims A.H., Briegel K.J. (2012). The T Box Transcription Factor TBX2 Promotes Epithelial-Mesenchymal Transition and Invasion of Normal and Malignant Breast Epithelial Cells. PLoS ONE.

[57] Lamouille S., Xu J., Derynck R. (2014). Molecular Mechanisms of Epithelial–Mesenchymal Transition. Nat. Rev. Mol. Cell Biol..

[58] Dyer L., Lockyer P., Wu Y., Saha A., Cyr C., Moser M., Pi X., Patterson C. (2015). BMPER Promotes Epithelial-Mesenchymal Transition in the Developing Cardiac Cushions. PLoS ONE.

[59] Zhu F., Duan Y.-F., Bao W.-Y., Liu W.-S., Yang Y., Cai H.-H. (2015). HtrA1 Regulates Epithelial–Mesenchymal Transition in Hepatocellular Carcinoma. Biochem. Biophys. Res. Commun..

[60] Frantz C., Stewart K.M., Weaver V.M. (2010). The Extracellular Matrix at a Glance. J. Cell Sci..

[61] Sun X., Malandraki-Miller S., Kennedy T., Bassat E., Klaourakis K., Zhao J., Gamen E., Vieira J.M., Tzahor E., Riley P.R. (2021). The Extracellular Matrix Protein Agrin Is Essential for Epicardial Epithelial-to-Mesenchymal Transition during Heart Development. Development.

[62] Wang J., Karra R., Dickson A.L., Poss K.D. (2013). Fibronectin Is Deposited by Injury-Activated Epicardial Cells and Is Necessary for Zebrafish Heart Regeneration. Dev. Biol..

[63] Ramjee V., Li D., Manderfield L.J., Liu F., Engleka K.A., Aghajanian H., Rodell C.B., Lu W., Ho V., Wang T. (2017). Epicardial YAP/TAZ Orchestrate an Immunosuppressive Response Following Myocardial Infarction. J. Clin. Investig..

[64] Cao J., Wang J., Jackman C.P., Cox A.H., Trembley M.A., Balowski J.J., Cox B.D., De Simone A., Dickson A.L., Di Talia S. (2017). Tension Creates an Endoreplication Wavefront That Leads Regeneration of Epicardial Tissue. Dev. Cell.

[65] Tang T., Li L., Tang J., Li Y., Lin W.Y., Martin F., Grant D., Solloway M., Parker L., Ye W. (2010). A Mouse Knockout Library for Secreted and Transmembrane Proteins. Nat. Biotechnol..

[66] Runyan R.B., Markwald R.R. (1983). Invasion of Mesenchyme into Three-Dimensional Collagen Gels: A Regional and Temporal Analysis of Interaction in Embryonic Heart Tissue. Dev. Biol..

[67] Livak K.J., Schmittgen T.D. (2001). Analysis of Relative Gene Expression Data Using Real-Time Quantitative PCR and the 2^−ΔΔCT^ Method. Methods.

[68] Dobin A., Gingeras T.R. (2015). Mapping RNA-seq Reads with STAR. Curr. Protoc. Bioinform..

[69] Liao Y., Wang J., Jaehnig E.J., Shi Z., Zhang B. (2019). WebGestalt 2019: Gene set analysis toolkit with revamped UIs and APIs. Nucleic Acids Res..

[70] McCarthy D.J., Chen Y., Smyth G.K. (2019). Differential expression analysis of multifactor RNA-Seq experiments with respect to biological variation. Nucleic Acids Res..

